# Lateromedial and oblique radiographs detect most fetlock pathologies as effectively as a full series in horses

**DOI:** 10.1111/evj.70073

**Published:** 2025-08-13

**Authors:** A. Northwood, D. Berner

**Affiliations:** ^1^ The Royal Veterinary College Equine Referral Hospital Hertfordshire UK

**Keywords:** horse, interobserver, intraobserver, metacarpophalangeal, metatarsophalangeal, pre‐purchase

## Abstract

**Background:**

Radiographic protocols for the metacarpo‐/tarsophalangeal joint during pre‐purchase examinations (PPE) vary internationally, but their impact on pathology detection remains unclear. Optimising imaging protocols is essential to balance diagnostic accuracy with workflow efficiency and radiation exposure.

**Objectives:**

To evaluate the effect of different radiographic view combinations on fetlock pathology detection and observer agreement in a PPE context; hypothesising that detection rates vary with view selection.

**Study Design:**

Retrospective observational study.

**Methods:**

Two observers reviewed fetlock radiographic series using four view combinations: lateromedial (LM) alone, LM and dorsopalmar/plantar (LM/DP), LM and oblique projections (LM/OB), and the full series (FULL). McNemar's chi‐squared test assessed detection differences; Cohen's kappa evaluated intra‐ and inter‐observer agreement, and diagnostic parameters were calculated for reduced views relative to the full series.

**Results:**

A total of 673 fetlock series were reviewed. The LM/OB combination showed no significant difference in detecting most pathologies compared to the full series. Observer agreement was generally highest with the full series. Intra‐observer agreement was highest for LM/OB, except for subchondral bone changes in the proximal phalanx, where LM/DP performed better; though overall agreement was low. Lateromedial projections reliably detected fragmentation, sesamoid fractures, and osseous cyst‐like lesions. Oblique views were superior for sesamoid bone changes.

**Main Limitations:**

Retrospective design, selection bias, and lack of gold standard confirmation.

**Conclusions:**

While LM/OB offers a practical compromise in many PPE scenarios, certain pathologies, particularly subchondral bone changes, may require additional views. A tailored approach based on age, discipline, and clinical risk may optimise diagnostic yield.

## Abstract (fr)

**Background:**

Les protocoles radiographiques pour l’articulation métacarpo‐/tarsophalangienne durant les examens vente et achat varient d’un pays à l’autre et leur impact sur la détection des pathologies demeure incertain. L’optimisation des protocoles d’imagerie reste essentielle afin de balancer l’acuité diagnostique avec l’exposition aux radiations et l’efficacité de travail.

**Objectifs:**

Évaluer l’effet de différentes combinaisons de projections radiographiques sur la détection des pathologies du boulet équin et le degré d’accord des observateurs dans un contexte d’examen vente et achat, avec l’hypothèse que les taux de détection varient selon la projection sélectionnée.

**Type d’étude:**

Étude rétrospective observationnelle.

**Méthodes:**

Deux observateurs ont examiné des séries de radiographies du boulet en utilisant 4 combinaisons de projections: latéromédiale uniquement (LM), LM et dorsopalmaire/plantaire (LM/DP), LM et projections obliques (LM/OB) et séries complètes (FULL). Le test du khi carré de McNemar a été utilisé pour analyser les différences de détection, le Kappa de Cohen a évalué l’accord inter‐intra‐observateur et les paramètres diagnostics ont été calculés pour les combinaisons de projections comparativement aux séries complètes.

**Résultats:**

Un total de 673 séries radiographiques du boulet ont été examinées. La combinaison LM/OB n’a montré aucune différence dans la détection de la majorité des pathologies, comparativement aux séries complètes. L’accord des observateurs était généralement supérieur avec les séries complètes. L’accord intra‐observateur était plus élevé pour les LM/OB, à l’exception des changements de l’os sous‐chondral au niveau de la première phalange, où les LM/DP étaient plus diagnostiques, tous avec un accord somme toute bas. Les projections latéromédiales ont détecté les fragmentations, les fractures des os sésamoïdiens proximaux et les lésions osseuses de forme kystique.

**Limitations principales:**

Nature rétrospective de l’étude, biais de sélection, manque de gold standard pour confirmation absolue.

**Conclusions:**

Alors que les LM/OB offre un compromis pratique pour plusieurs scénarios d’examen vente et achat, certaines pathologies, tel que les changements osseux sous‐chondraux, peuvent nécessiter davantage de projections radiographiques. Une approche adaptée à l’individu, basée sur l’âge, la discipline et les risques cliniques devrait optimiser le rendement diagnostic.

## 1 INTRODUCTION

In equine pre‐purchase examinations (PPE), radiographs are commonly used to detect pathology affecting future soundness. However, their value is debated due to concerns about radiation exposure and the variability of findings.^1^ The metacarpo‐/tarsophalangeal joint has become a key focus in radiographic evaluations due to the high prevalence of lesions.^2^ Studies from various disciplines indicate a variable impact of radiographic fetlock changes on performance.^3^, ^4^, ^5^, ^6^ These findings highlight that discipline and context influence how radiographic abnormalities correlate with performance.

This uncertainty is compounded by regional discrepancies in radiographic guidelines for PPEs. While some recommendations exist regarding optimal projections for identifying specific pathologies,^7^ these guidelines vary significantly across regions. For example, the German Equine Veterinary Association (GEVA) recommends only a lateromedial (LM) projection, with additional views taken only if indicated.^8^ In contrast, the Netherlands employs a combination of LM and oblique projections in the forelimb—specifically, the dorsolateral‐palmaromedial oblique (DLPMO) and dorsomedial‐palmarolateral oblique (DMPLO), but only a single LM and a recently added dorsoplantar (DP) projection for the hindlimb.^9^ In the United Kingdom, guidelines are more specific to racehorses, advising the LM, DP, and both oblique views (DLPMO and DMPLO), with an optional flexed LM projection for the forelimb.^10^ However, no formal guidelines exist for non‐racehorses, though a more comprehensive series, typically including LM, DP, and both oblique views, is commonly used in practice. These regional differences reflect a broader lack of consensus on the most effective radiographic protocols for detecting performance‐related pathologies. This uncertainty is further highlighted by similar studies in both human and small animal species, specifically in canine thoracic imaging, which have aimed to reduce the number of views but yielded inconsistent results.^11^, ^12^, ^13^ While these studies are in different anatomical and clinical contexts, such variability underscores the ongoing debate surrounding optimal radiographic protocols and their application in clinical practice.

No study has definitively identified the optimal single projection or combination of projections to detect most pathologies in the fetlock region while minimising radiation exposure. This study aimed to determine the most effective projection or combination, balancing accuracy and radiation exposure. Therefore, inter‐observer and intra‐observer agreement, and diagnostic metrics (sensitivity, specificity, positive predictive value [PPV], and negative predictive value [NPV]) of reduced view combinations were assessed compared to the full series. The hypotheses were: (1) there would be a significant difference in pathology detection across projection combinations; (2) variability in observer agreement would occur depending on the combination used; and (3) increasing the number of projections would improve diagnostic accuracy.

## 2 MATERIALS AND METHODS

### 2.1 Population

**FIGURE 1:**
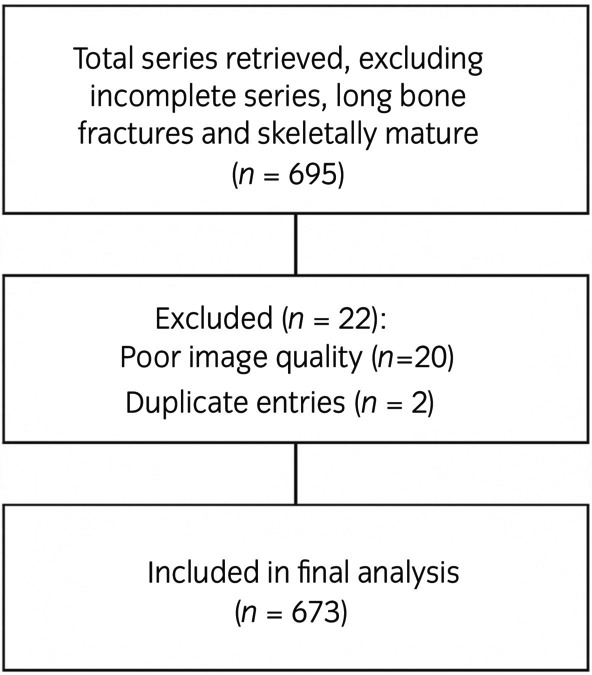
Flowchart illustrating case selection. A total of 695 radiographic series were retrieved from the archives after excluding cases with incomplete series, long bone fractures, or skeletally mature horses. Of these, 22 cases were excluded due to poor image quality (*n* = 20) or duplicate entries (*n* = 2), resulting in 673 series included in the final analysis.

Fetlock radiographic series were retrospectively collected from May 2013 to August 2023 from the Royal Veterinary College (RVC) archives (Figure 1). Due to the limited availability of true pre‐purchase radiographs, the study population predominantly comprised horses undergoing orthopaedic evaluations. Although not originally acquired for PPEs, the images were interpreted in a pre‐purchase examination context. Exclusion criteria were incomplete standard series, inappropriate labelling, long bone fractures, wounds, or skeletal immaturity. Where available, age, breed, and sex were recorded.

### 2.2 Radiographic evaluation

All radiographs were anonymised and randomised before review. Two independent observers (a diplomate and a resident of the European College of Veterinary Diagnostic Imaging) assessed the images using DICOM viewing software (Horos Project). Radiographs were reviewed in independent stages: (1) LM projection alone (LM); (2) LM and DP (LM/DP); (3) LM, DLPMO and DMPLO (LM/OB); and (4) the full standard series (FULL) of all the above. All DP and oblique projections were acquired with a 10–15‐degree proximodistal elevation relative to horizontal to better separate the proximal sesamoid bones from the joint surfaces.

Observers were instructed to evaluate the radiographs as though they were conducting a pre‐purchase examination. Diagnostic quality was assessed on a 1–5 scale, with 1 indicating perfect quality and 5 indicating a non‐diagnostic image. Any series containing an image rated as 4 or 5 was excluded from subsequent statistical analysis. Observers then recorded the presence or absence of pathology, grading common pathologies such as osteoarthritis on a pre‐determined system ranging from 0 (normal) to 3 (severe) adapted from previous studies (Table 1).^4^, ^14^

**TABLE 1:** Grading key for different pathologies of the fetlock joint.

Pathology	Score	Description
Osteoarthritic changes	0	No changes detected
	1	Mild regular modelling on the articular margins
	2	Moderate regular or mild irregular modelling on the articular margins
	3	Large, marked regular or moderate irregular modelling on the articular margins
Osteochondral changes	0	No changes detected
	1	Mild defect to the bone contour
	2	Moderate defect to the bone contour
	3	Marked defect to the bone contour
Sesamoid changes	0	No changes detected
	1	Increased linear defects within the sesamoid bones (<2 mm)
	2	Increased, slightly abnormal shaped defects within the sesamoid bones (<2 mm)
	3	Increased, abnormally shaped defects within the sesamoid bones (>2 mm)
Osseous cyst‐like lesions	0	No changes detected
	1	Mild, focal subchondral bone flattening
	2	Small, oval lucent area with surrounding sclerotic rim
	3	Large, oval lucent area with surrounding sclerotic rim
Subchondral bone changes	0	No changes detected
	1	Slight focal lucency in the subchondral bone plate (mild demineralisation)
	2	Marked lucency in the subchondral bone plate, possibly extending into underlying trabecular bone
	3	Extensive subchondral bone lucency with cortical thinning/collapse
Sesamoid fractures	0	No changes detected
	1	Present
Enthesopathy	0	No changes detected
	1	Mild bone changes at the site of tendon/ligament insertion
	2	Moderate bone changes at the site of tendon/ligament insertion
	3	Marked bone changes at the site of tendon/ligament insertion
Fragmentation	0	No fragment present
	1	Small fragment present
	2	Moderate sized fragment present
	3	Large fragment present

### 2.3 Data analysis

Data was recorded in a spreadsheet and analysed using SPSS software. Cohen's kappa statistic was employed to assess both intra‐ and inter‐observer agreement for the detection of pathology, while weighted kappa was utilised to evaluate agreement regarding the severity of pathology. Kappa values were interpreted as follows: poor (<0.20), fair (0.21–0.40), moderate (0.41–0.60), substantial (0.61–0.80), and excellent (>0.80) with a p value of <0.05. McNemar's chi‐squared test was applied to compare the detection of pathology across different projection combinations, with a p‐value greater than 0.05 indicating no significant difference between combinations. Findings considered normal or mild (grade 0/1) as well as moderate or severe pathology (grade 2/3) were later combined to determine whether statistical significance was also present between combinations of pathology. Specificity, sensitivity, positive predictive value (PPV), and negative predictive value (NPV) for each pathology were calculated for the different projection combinations using the full series of projections as the gold standard.

## 3 RESULTS

A total of 695 complete fetlock series were included and reviewed. After exclusion of 20 series for poor diagnostic quality and two duplicates, 673 series from 342 horses were included: 327 forelimb and 346 hindlimb series. The population included 132 mares, 114 geldings, and 71 stallions with a median age of 9 years (range 2–23 years). Sex was unrecorded in 25 cases whilst age was unrecorded in 36 horses. Horse breeds included Warmbloods (*n* = 95), Thoroughbreds (*n* = 62), cobs (*n* = 41), ponies (*n* = 20), draught breeds (*n* = 12), and Arabs (*n* = 4). The remaining 108 horses were a mix of other breeds, with 36 being unknown. Most series (*n* = 634; 94%) were obtained for lameness investigations, while 39 (6%) were routine pre‐purchase studies.

### 3.1 Findings

Observer 1 classified 39 series (5.8%, 95% Confidence Interval [CI] 4.14%–7.84%) as normal, while Observer 2 classified 32 series (4.8%, CI 3.2%–6.6%) as normal. Osteoarthritis was the most common finding, detected in 512 series (76.1%, CI 72.6%–79.2%) by Observer 1 and 437 series (64.9%, CI 61.2%–68.5%) by Observer 2. Many of these cases were graded as mild (55.6%, CI 51.7%–59.4% and 49.1%, CI 45.3%–53.0%, respectively); though osteoarthritis also had the highest proportion of severe ratings (1.6%, CI 0.82%–2.9%) for Observer 1 and (1%, CI 0.4%–2.1%) for Observer 2. Osteochondrosis was the second most frequent finding, identified in 397 series (59%, CI 55.1%–62.7%) by Observer 1 and 221 series (37%, CI 29.3%–36.5%) by Observer 2. Enthesopathy of the proximal phalanx and sesamoid changes were also commonly detected. Less common findings included dorsal and palmar/plantar fragmentation, subchondral bone changes of the proximal phalanx and third metacarpal/metatarsal bone, osseous cyst‐like lesions (OCLLs), and sesamoid bone abnormalities.

### 3.2 McNemar's chi‐squared

No statistically significant differences were found in the detection of dorsal, palmar, or plantar fragmentation between any projection combinations compared to the full series, except for dorsal fragmentation in the lateromedial view for Observer 1 (*p* > 0.05). Similarly, only one combination (LM for Observer 2) showed a significant difference in the detection of proximal sesamoid bone fractures and OCLLs of the proximal phalanx and third metacarpal bone, but again, only for one observer on each occasion.

Generally, LM/OB showed the fewest statistical differences when compared to the full series for the detection of pathologies. No significant differences were noted in categories such as enthesopathy or sesamoid fractures for both observers. Notably, LM/OB consistently maintained diagnostic accuracy across a range of pathologies. A detailed summary of these findings is provided in Table 2.

**TABLE 2:** McNemar's chi‐squared for the detection of pathology.

	LM		LM/DP		LM/OB	
	Obs 1	Obs 2	Obs 1	Obs 2	Obs 1	Obs 2
Osteoarthritis						X
Osteochondrosis						
Dorsal fragment		X	X	X	X	X
Pa/Pl fragment	X	X	X	X	X	X
Enthesopathy					X	X
OCLL (P1)		X	X	X	X	X
OCLL (MC/MTIII)	X		X	X	X	X
Subchondral bone change (P1)				X		X
Subchondral bone change (MC/MTIII)						
Sesamoid changes						
Sesamoid fractures	X		X	X	X	X

*Note*: Combinations were compared against the full series. ‘X’ represents no statistical difference (*p* > 0.05).

Abbreviations: LM, single lateromedial view; LM/DP, lateromedial and dorsopalmar(plantar) view; LM/OB, lateromedial, dorsomedial‐palmaro(plantaro)lateral oblique and dorsolateral‐palmaro(plantaro)lateral oblique; MC/MT III, third metacarpal/tarsal bone; Obs 1, observer 1; Obs 2, observer 2; OCLL, osseous cyst like lesion; P1, proximal phalanx; Pa, palmar; Pl, plantar.

When analysis was limited to moderate (grade 2) and severe (grade 3) cases, no statistically significant differences were found between LM/OB and FULL for the detection of any pathology with Observer 2. Observer 1 demonstrated higher detection rates for moderate to severe cases in LM/OB compared to when mild (grade I) cases were included in the analysis. Table 3 provides further details on the detection of moderate to severe pathology.

**TABLE 3:** McNemar's chi‐squared for the detection of moderate or severe pathology.

	LM		LM/DP		LM/OB	
	Obs 1	Obs 2	Obs 1	Obs 2	Obs 1	Obs 2
Osteoarthritis					X	X
Osteochondrosis		X			X	X
Dorsal fragment	X	X	X	X	X	X
Pa/Pl fragment	X	X	X	X	X	X
Enthesopathy				X	X	X
OCLL (P1)		X	X	X	X	X
OCLL (MC/MTIII)	X		X	X	X	X
Subchondral bone change (P1)			X	X		X
Subchondral bone change (MC/MTIII)		X		X		X
Sesamoid changes						X
Sesamoid fractures	X		X	X	X	X

*Note*: Combinations were compared against the full series. ‘X’ represents no statistical difference (*p* > 0.05).

Abbreviations: LM, single lateromedial view; LM/DP, lateromedial and dorsopalmar(plantar) view; LM/OB, lateromedial, dorsomedial‐palmaro(plantaro)lateral oblique and dorsolateral‐palmaro(plantaro)lateral oblique; MC/MT III, third metacarpal/tarsal bone; Obs 1, Observer 1; Obs 2, Observer 2; OCLL, osseous cyst like lesion; P1, proximal phalanx; Pa, palmar; Pl, plantar.

### 3.3 Intra‐observer agreement

Table 4 summarises the intra‐observer agreement for the detection of individual pathologies compared to the full series. Overall, agreement levels improved with the inclusion of more projections. Sesamoid fractures and fetlock joint fragmentation demonstrated substantial to excellent intra‐observer agreement across all projection combinations, with kappa values ranging from 0.61 to 0.87 for sesamoid fractures and 0.65 to 0.89 for fetlock joint fragmentation. For almost all other pathologies, LM/OB generally provided the highest intra‐observer agreement. A similar trend was seen in all pathologies when looking at moderate or severe cases only, with some minor improvements in agreement noted. The exception to this was subchondral bone changes in the proximal phalanx showing the best agreement in the LM/DP combination. Despite this, agreement remained poor across all combinations for this pathology, with kappa values below 0.2 and some combinations yielding non‐significant results (*p* > 0.05). Similar levels of non‐significant and poor to moderate agreement were observed for subchondral bone changes in the third metacarpal/metatarsal bone.

**TABLE 4:** Intra‐observer agreement for the detection of individual pathology compared to the full series (*p* < 0.05).

	Observer 1			Observer 2		
	LM	LM/DP	LM/OB	LM	LM/DP	LM/OB
Osteoarthritis	0.26 (0.17–0.34)	0.29 (0.21–0.37)	0.52 (0.44–0.60)	0.23 (0.17–0.30)	0.33 (0.26–0.40)	0.64 (0.59–0.71)
Osteochondrosis	0.34 (0.27–0.40)	0.37 (0.30–0.44)	0.48 (0.44–0.55)	0.45 (0.38–0.52)	0.41 (0.33–0.49)	0.53 (0.46–0.60)
Dorsal fragment	0.65 (0.53–0.78)	0.71 (0.58–0.84)	**0.81 (0.71–0.91)**	**0.81 (0.70–0.92)**	**0.83 (0.74–0.86)**	**0.89 (0.81–0.97)**
Pa/Pl fragment	0.79 (0.67–0.91)	**0.88 (0.78–0.98)**	**0.89 (0.79–0.98)**	0.70 (0.56–0.84)	0.76 (0.63–0.88)	0.83 (0.73–0.93)
Enthesopathy	0.07 (0.00–0.15)	0.06 (0.05–0.10)	0.70 (0.64–0.75)	0.09 (0.00–0.18)	0.21 (0.12–0.29)	0.67 (0.61–0.72)
Subchondral bone change (P1)	0.05 (0.00–0.20)	0.17 (0.03–0.30)	0.06 (0.00–0.22)	n.s.	0.18 (0.07–0.29)	0.18 (0.06–0.29)
Subchondral bone change (MC/MTIII)	n.s.	0.17 (0.10–0.25)	0.26 (0.18–0.33)	n.s.	0.30 (0.23–0.38)	0.51 (0.44–0.58)
Sesamoid changes	0.09 (0.02–0.15)	0.13 (0.06–0.20)	0.49 (0.42–0.57)	0.11 (0.04–0.17)	0.18 (0.11–0.24)	0.44 (0.37–0.51)
Sesamoid fractures	**0.86 (0.66–1.00)**	**0.86 (0.69–1.00)**	**0.87 (0.70–1.00)**	0.61 (0.37–0.85)	0.70 (0.51–0.90)	0.75 (0.59–0.91)

*Note*: Not significant values are represented as n.s. Values higher than 0.8 are in bold. Confidence intervals (95%) are in parentheses.

Abbreviations: LM, single lateromedial view; LM/DP, lateromedial and dorsopalmar(plantar) view; LM/OB, lateromedial, dorsomedial‐palmaro(plantaro)lateral oblique and dorsolateral‐palmaro(plantaro)lateral oblique; Pa, palmar; Pl, plantar; P1, proximal phalanx; MC/MT III, third metacarpal/tarsal bone.

### 3.4 Inter‐observer agreement

Inter‐observer agreement for the detection of various pathologies across the different projection combinations varied from low to high (Table 5). The highest agreement was observed for fragmentation (both dorsal and palmar/plantar), sesamoid bone fractures, and osteoarthritis, particularly with FULL and LM/OB, where kappa values ranged from 0.35 to 0.79. The full series generally provided the most reliable results, with LM/OB also demonstrating good consistency. In contrast, LM/DP showed more variability, with lower kappa values for pathologies such as enthesopathy and sesamoid bone changes. Overall, the full series and LM/OB offered the most consistent inter‐observer agreement across all pathologies, while LM/DP were less reliable for certain conditions.

**TABLE 5:** Inter‐observer agreement for the detection of individual pathology (*p* ≤ 0.05).

	LM	LM/DP	LM/OB	FULL
Osteoarthritis	0.26 (0.19–0.34)	0.39 (0.32–0.46)	0.43 (0.36–0.49)	0.53 (0.46–0.59)
Osteochondrosis	0.30 (0.23–0.37)	0.24 (0.17–0.32)	0.30 (0.23–0.37)	0.35 (0.28–0.42)
Dorsal fragment	0.74 (0.69–0.79)	0.78 (0.73–0.82)	0.76 (0.71–0.81)	0.76 (0.72–0.81)
Pa/Pl fragment	0.78 (0.73–0.82)	**0.81 (0.77–0.86)**	**0.83 (0.78–0.87)**	0.75 (0.70–0.80)
Enthesopathy	0.33 (0.26–0.40)	0.18 (0.11–0.25)	0.66 (0.60–0.71)	0.67 (0.61–0.72)
Sesamoid changes	0.14 (0.07–0.22)	0.32 (0.24–0.39)	0.40 (0.34–0.47)	0.70 (0.65–0.76)
Sesamoid fractures	0.80 (0.75–0.84)	0.70 (0.64–0.75)	0.56 (0.50–0.62)	0.55 (0.49–0.61)

*Note*: Values higher than 0.8 are in bold. Confidence intervals (95%) are in parentheses.

Abbreviations: FULL, full standard radiographic series; LM, single lateromedial view; LM/DP, lateromedial and dorsopalmar(plantar) view; LM/OB, lateromedial, dorsomedial‐palmaro(plantaro)lateral oblique and dorsolateral‐palmaro(plantaro)lateral oblique.

### 3.5 Sensitivity, specificity, positive predictive value (PPV) and negative predictive value (NPV)

Diagnostic accuracy parameters of three reduced radiographic combinations were reviewed for various pathologies, using the results of the more experienced observer outlined in Table 6. Diagnostic parameters for osseous cyst‐like lesions could not be completed due to low case numbers. Overall, for most pathologies, sensitivity and NPV increased with the increasing number of views available, whilst specificity decreased, and PPV remained mostly static.

**TABLE 6:** Sensitivity, specificity, positive and negative predictive value of the different combinations when compared to the full series.

	Sensitivity (%)			Specificity (%)			PPV (%)			NPV (%)		
	LM	LM/DP	LM/OB	LM	LM/DP	LM/OB	LM	LM/DP	LM/OB	LM	LM/DP	LM/OB
Osteoarthritis	39.13 (34.53–43.88)	62.70 (57.98–67.25)	**88.79 (85.45–91.59)**	**89.83 (85.25–93.37)**	73.73 (67.63–79.23)	79.66 (73.95–84.61)	**87.69 (82.73–91.38)**	**81.55 (77.91–84.70)**	**88.99 (86.24–91.25)**	44.35 (42.23–46.50)	51.63 (48.05–55.20)	79.32 (74.52–83.43)
Osteochondrosis	43.83 (38.88–48.87)	52.39 (47.35–57.40)	70.53 (65.78–74.97)	**93.48 (89.89–96.06)**	**87.68 (83.21–91.32)**	**82.84 (78.39–86.70)**	**90.63 (85.95–93.87)**	**85.95 (81.50–89.47)**	**82.84 (79.11–86.02)**	53.64 (51.34–55.92)	56.15 (53.37–58.89)	70.53 (67.10–73.74)
Dorsal fragment	**82.35 (65.47–93.24)**	73.53 (55.64–87.12)	**88.24 (72.55–96.70)**	**96.71 (95.02–97.95)**	**98.44 (97.12–99.25)**	**98.59 (97.34–99.25)**	57.14 (45.00–67.62)	71.43 (56.69–82.68)	76.92 (63.27–86.58)	**99.04 (98.03–99.53)**	**98.59 (97.56–99.19)**	**98.59 (98.43–99.75)**
Pa/Pl fragment	78.57 (59.05–91.70)	**82.14 (63.11–93.94)**	**89.29 (71.77–97.73)**	**81.48 (61.92–93.70)**	**99.53 (98.65–99.90)**	**99.53 (98.65–99.90)**	**81.48 (64.27–91.50)**	**88.46 (70.99–96.00)**	**89.29 (72.79–96.29)**	**99.07 (98.13–99.54)**	**99.23 (98.31–99.65)**	**99.53 (98.66–99.84)**
Enthesopathy	9.66 (6.52–13.65)	9.60 (4.26–10.45)	**80.34 (75.30–84.76)**	**96.87 (94.59–98.37)**	**98.43 (96.62–99.42)**	**88.77 (85.18–91.75)**	**70.00 (54.70–81.85)**	76.92 (57.55–89.12)	**84.42 (80.26–87.84)**	58.61 (57.59–59.62)	58.27 (57.45–59.09)	**85.64 (82.50–88.30)**
Subchondral bone change (P1)	8.33 (3.88–15.23)	21.30 (14.00–30.22)	6.48 (2.65–12.90)	**95.22 (93.12–96.83)**	**92.57 (90.08–94.59)**	**97.70 (96.10–98.77)**	25.00 (13.89–40.79)	35.38 (13.35–19.04)	35.00 (18.03–56.87)	**84.46 (83.66–85.23)**	**86.02 (84.76–87.19)**	**84.53 (80.01–85.82)**
Subchondral bone change (MC/MTIII)	24.24 (19.72–29.24)	40.30 (34.97–45.81)	56.36 (50.82–61.79)	**80.17 (75.56–84.26)**	76.68 (71.84–81.05)	69.39 (64.21–74.22)	54.05 (46.92–61.02)	62.44 (56.85–67.72)	63.92 (59.54–68.08)	52.38 (50.37–54.39)	57.17 (54.56–59.75)	62.30 (58.93–65.56)
Sesamoid changes	34.47 (30.17–38.96)	48.72 (44.12–53.35)	**91.06 (88.11–93.48)**	77.34 (70.96–82.91)	69.46 (62.62–75.71)	55.17 (48.05–62.12)	77.88 (72.63–82.38)	78.69 (74.64–82.26)	**82.47 (80.11–84.60)**	33.76 (31.58–36.02)	36.91 (34.01–39.91)	72.73 (66.08–78.50)
Sesamoid fractures	**85.71 (42.13–99.64)**	**85.71 (42.13–99.64)**	**100 (59.04–100.00)**	**99.58 (99.17–100.00)**	**99.58 (99.17–100)**	**99.70 (98.92–99.96)**	**85.71 (45.26–97.75)**	**85.71 (45.26–97.75)**	77.78 (46.73–93.32)	**99.85 (99.09–99.98)**	**99.85 (99.09–99.98)**	**100 (99.45–100.00)**

*Note*: Confidence intervals (95%) are in parentheses. Values higher than 80% are in bold.

Abbreviations: LM/DP, lateromedial and dorsopalmar(plantar) view; LM/OB, lateromedial, dorsomedial‐palmaro(plantaro)lateral oblique and dorsolateral‐palmaro(plantaro)lateral oblique; LM, single Lateromedial view; MC/MT III, third metacarpal/tarsal bone; NPV, negative predictive value; P1, proximal phalanx; Pa, palmar; Pl, plantar; PPV, positive predictive value.

The LM/OB combination consistently demonstrated the highest sensitivity for most conditions, including osteoarthritis (88.79%, CI 85.45%–91.59%), osteochondrosis (70.53%, CI 65.78%–74.97%), dorsal fragments (88.24%, CI 75.55%–96.70%), palmar/plantar fragments (89.29%, CI 71.77%–97.73%), and sesamoid fractures (100%, CI 59.04%–100%). The LM/DP combination provided intermediate sensitivity, outperforming LM for conditions such as osteochondrosis (52.39%, CI 47.35%–57.40%) and palmar/plantar fragments (82.14%, CI 63.11%–93.94%). In contrast, LM showed notably lower sensitivity across most pathologies, except for certain subchondral bone changes in P1 and MC/MTIII, where all combinations performed poorly.

Specificity was generally high for LM and LM/DP across most conditions, peaking at 99.53% (CI 98.65%–99.90%) for palmar/plantar fragments with both combinations. On the other hand, LM/OB exhibited lower specificity for some conditions, such as osteoarthritis (79.66%, CI 73.95%–84.61%) and osteochondrosis (82.84%, CI 78.39%–86.70%), while maintaining high specificity for fractures and subchondral bone changes. Positive predictive value was highest for LM/OB in detecting palmar/plantar fragments (89.29%, CI 72.79%–96.29%), dorsal fragments (76.92%, CI 63.27%–86.58%), and osteoarthritis (88.99%, CI 86.24%–91.25%). Similarly, LM/OB achieved high NPV, particularly for sesamoid fractures (100%, CI 99.45%–100%) and dorsal fragments (98.59%, CI 98.43%–99.75%).

Certain pathology‐specific observations were notable. Enthesopathy detection had extremely low sensitivity with LM and LM/DP (9.66%–9.60%) but improved dramatically with LM/OB (80.34%, CI 75.30%–84.76%). For fragmentation and sesamoid bone fractures, although diagnostic parameters slightly changed, they mainly remained static across the different combinations and were uniformly well identified, with LM/OB achieving 100% sensitivity, specificity, and NPV.

## 4 DISCUSSION

This study evaluates the diagnostic efficacy of reduced radiographic projection combinations for detecting fetlock pathologies. This has important implications for equine pre‐purchase examinations (PPE), which are a common source of equine‐related disputes in the UK.^15^ While radiographic protocols must adhere to the ALARA (As Low As Reasonably Achievable) radiation protection principle, by comparing reduced radiographic combinations to a full series, our findings indicate that the LM/OB combination delivers comparable diagnostic performance to the full four‐view series across a broad range of pathologies, optimising diagnostic accuracy while reducing cost and radiation exposure, making it a satisfactory alternative for routine PPEs in sports and pleasure horses. However, additional views such as the DP may still be warranted for horses with higher performance demands or discipline‐specific vulnerabilities, such as Thoroughbred racehorses. Reducing the number of projections from four to three results in approximately a 25% reduction in per fetlock exam radiation exposure. Although the per‐image dose is minimal with modern systems, reducing the number of projections supports the ALARA principles and helps limit cumulative radiation exposure over time.

The first hypothesis, that pathology detection would vary across projection combinations, was partially supported. The LM/OB combination showed no significant differences in detection rates for most pathologies compared to the full series, suggesting that reduced protocols may be sufficient, though more comprehensive imaging may still be warranted for certain findings, such as subchondral bone changes. A similar trend has been observed in fracture detection in the canine tarsus, where two‐view and ten‐view radiographic studies demonstrated similar sensitivity and specificity, suggesting minimal benefit in additional views.^16^ Conversely, studies in companion animals' thoracic radiographs comparing two versus three views have yielded mixed results. While some studies have indicated that additional views improved accuracy,^11^, ^12^ others found no difference.^13^ For example, Ober and Barber (2006) found that the diagnosis of structured interstitial patterns in dogs changed in 12–15% of cases when increasing from two to three views.^11^ However, this difference may be more significant in the thoracic cavity, where lung compression can obscure lesions.^17^ Similarly, studies in human medicine, such as those comparing two‐ and three‐view radiographic protocols for diagnosing skull fractures, found no significant differences in diagnostic accuracy or interpretation confidence.^18^ While anatomical and clinical contexts differ, these findings suggest that reduced‐view protocols may be sufficient in certain situations without compromising diagnostic reliability.

Our study supports that while the full series of projections remains the gold standard for diagnostic agreement, the LM/OB combination offers comparable detection rates for most pathologies. The LM projection alone reliably detects pathologies such as fragmentation, sesamoid bone fractures, and osseous cyst‐like lesions, aligning with previous studies that suggest these projections are optimal for recognising these conditions.^19^ Adding oblique projections (DLPMO and DMPLO) further enhanced pathology detection, particularly for subtle sesamoid bone changes that were not easily detected with LM projections alone. This finding is consistent with previous literature emphasising the value of oblique projections for identifying sesamoid bone pathology.^20^ This is particularly relevant given the disparity in prevalence in horse populations. For instance, in a radiographic survey of 676 Warmblood stallions, no sesamoid fractures and only a single case of suspected sesamoiditis (0.15%) were reported.^21^ In contrast, studies in racehorses and high‐impact disciplines report much higher rates of sesamoid abnormalities, with up to 65% in young Thoroughbreds and 70% in lame barrel racing Quarter Horses.^22^, ^23^ These differences suggest that the prevalence of sesamoid‐related pathology in PPEs may be discipline‐specific. Overall, whilst the LM alone appears sufficient for some conditions, the LM/OB combination is a practical and reliable alternative to the full series for many fetlock pathologies.

**FIGURE 2:**
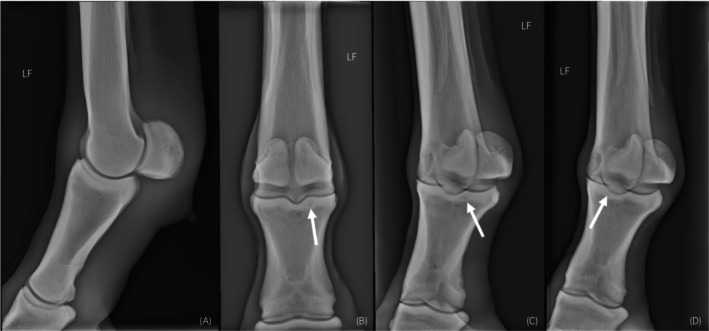
Full series of radiographs of the metacarpophalangeal joint from one horse, demonstrating a subchondral bone lucency (white arrows) located just lateral to the sagittal groove. (A) Lateromedial (LM) view: The lucency is not appreciable. (B) Dorsopalmar (DP) view: The lucency is visible. (C) Dorsolateral–palmaromedial oblique (DLPMO) view: The lucency is identifiable but less distinct. (D) Dorsomedial–palmarolateral oblique (DMPLO) view: The lucency is only subtly visible and may be overlooked without information from the other views. This case highlights the diagnostic value of the dorsopalmar projection for detecting subchondral lucencies in this location.

In contrast, evaluating subchondral bone changes in the proximal phalanx proved more challenging. Though the LM/DP combination improved detection, observer agreement was low, suggesting inconsistent identification of these lesions. An example of a more readily appreciable lesion is shown in Figure 2. This likely reflects the subtlety of early subchondral changes, which often lack sufficient radiopacity for clear differentiation from normal variations. Furthermore, overlapping anatomical structures can obscure detail, reducing detection accuracy, particularly on the DP projection.^24^ Observer‐dependent factors, such as training and interpretive thresholds, also contribute to variability, as these changes often require subjective pattern recognition. Specialised projections particularly useful in at‐risk groups, such as the flexed dorsopalmar/plantar view, have also been described to help identify these lesions but still tend to underestimate them.^25^, ^26^ Additionally, horses with significant subchondral bone pathology are often clinically lame and may not reach the radiographic stage of a pre‐purchase examination, underrepresenting these lesions in PPE studies. However, it should be noted that lameness in these horses is often cyclical, with clinical signs temporarily resolving after short periods of rest.^27^ Given the clinical relevance of subchondral bone injury, particularly in performance horses, omitting projections such as the DP may risk missing significant lesions, underscoring the importance of tailoring protocols to the horse's discipline and risk profile. While computed tomography (CT) and magnetic resonance imaging (MRI) offer superior detection of subtle subchondral pathology,^28^, ^29^ their use in PPE evaluations remains limited due to cost considerations. Future studies comparing radiographic findings with CT‐confirmed subchondral bone defects could clarify the sensitivity of reduced‐view protocols in detecting clinically relevant lesions.

When moderate and severe cases of pathology were grouped to assess detection, the LM/OB combination demonstrated comparable diagnostic efficacy to the full series. One observer noted no statistical difference in detection rates across any of the pathology categories within this subgroup. Grouping higher‐grade lesions allowed evaluation of changes more likely to impact clinical decision‐making, as mild findings are often of uncertain or incidental significance. While moderate and severe pathologies are often linked to future soundness issues, radiographic findings do not always correlate with clinical signs.^4^ For instance, horses with dorsoproximal articular chip fractures, sesamoid bone fractures, or advanced sesamoiditis had lower odds of racing success, whereas conditions like enthesopathy showed no such link.^30^ This may suggest that enthesopathy is often clinically irrelevant in isolation. In our study, the LM/OB combination reliably detected the pathologies most associated with compromised performance, except for mild or equivocal sesamoid bone changes. In this study, ‘sesamoid changes’ encompassed a spectrum of alterations including increased vascular channels, marginal irregularities and sclerosis. Not all of these are diagnostic of sesamoiditis defined as the ‘variability in the radiological appearance of bony canals or channels within the equine proximal sesamoid bones’ and these may reflect normal physiological variation.^4^ As such, care should be taken when interpreting minor sesamoid changes in asymptomatic horses. In our study, subtle subchondral bone changes and equivocal sesamoid bone variations were most frequently missed findings in reduced‐view combinations.

**FIGURE 3:**
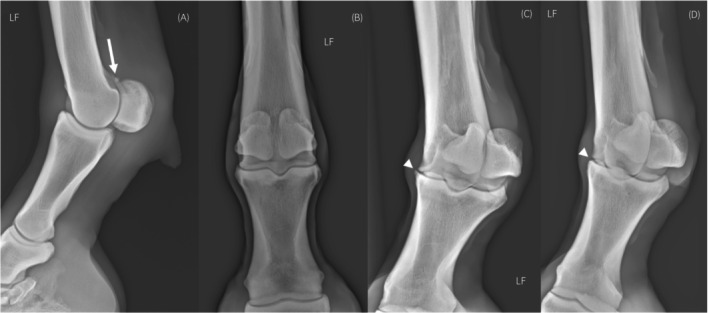
Full series of radiographs of the metacarpophalangeal joint from one horse, demonstrating different findings on various projections. (A) Lateromedial (LM) view showing a well‐defined, circular radiopacity indicative of a chronic sesamoid bone fracture (white arrow). (B) Dorsopalmar (DP) view. (C) Dorsolateral‐palmaromedial oblique (DLPMO) view, and (D) Dorsomedial‐palmarolateral oblique (DMPLO) view, both highlighting moderate osteoarthritic changes (white arrowheads), which were not detected in the LM or DP views.

Our second hypothesis that observer agreement would vary depending on the combination under review was partially supported. Both intra‐ and inter‐observer agreement generally improved with additional projections, but the magnitude of this improvement was condition dependent. While perfect agreement is rarely achieved in radiographic interpretation, kappa values above 0.60 are generally considered to represent substantial agreement and a reasonable benchmark for clinical reliability in previous radiographic studies.^31^ In this study, several projection combinations, particularly LM/OB, met or approached this threshold for common pathologies, supporting their potential utility in pre‐purchase settings. Pathologies with clear radiographic abnormalities, such as sesamoid fractures, showed high agreement even in reduced‐view combinations. This is expected, as certain pathologies are best appreciated on specific projections. For example, sesamoid bone disease is best assessed on oblique views due to reduced superimposition.^7^ Similarly, osteophytes in the fetlock typically first develop on the proximomedial aspect of the proximal phalanx, meaning early changes are most evident on oblique views (Figure 3).^7^ Conversely, subchondral bone changes exhibited low agreement across all projection combinations, likely due to their subtle nature of alterations and their dependency on factors like beam angle, image quality, and early‐stage bone remodelling. Differences in diagnostic threshold between observers likely also contributed to this variability. A similar trend was observed in the detection of osteochondrosis affecting the sagittal ridge of the third metacarpal/tarsal bone, where large discrepancies in detection rates between observers were evident—again likely reflecting interpretative variability. However, it should be noted that observer agreement can vary significantly for radiographs, independent of the number of views available.^32^

Our results suggest that age‐ or discipline‐related considerations may influence the optimal choice of radiographic projections during a PPE. In younger horses, particularly those being assessed for juvenile osteoarthritis or osteochondrosis, LM/OB projections may be sufficient to detect relevant pathology. However, in older performance horses such as show jumpers or dressage horses, inclusion of a DP view can be particularly valuable for assessing the integrity of the subchondral bone plate, which is more commonly affected in this group. Tailoring the radiographic protocol to the horse's age and intended use may therefore enhance diagnostic yield.

A key finding was the trade‐off between diagnostic accuracy and projection quantity. While our hypothesis that more projections would improve diagnostic accuracy was partially supported, with the additional projections improving sensitivity and NPV, specificity declined, increasing the risk of overdiagnosis. In PPE, missed pathologies can affect a horse's soundness, while overinterpretation could unjustly exclude suitable horses. Furthermore, variability in radiographic interpretation is well documented, as demonstrated by Esselman et al., who found that different practitioners assigned varying grades to the same findings.^33^ Practitioners must interpret radiographs within the broader clinical context, considering that mild changes, like early osteoarthritis, may not predict poor performance but could raise concerns for buyers. Radiographs should be integrated with clinical findings and the horse's intended use.

The main limitation of the current study was the absence of a gold standard comparison, such as advanced imaging techniques or histopathology, which could provide a definitive measure of diagnostic accuracy. The true diagnostic accuracy of these reduced view combinations remains uncertain without a direct comparison to a gold standard. Advanced imaging modalities, such as CT, have been shown to offer superior detection of pathologies in the fetlock region compared to traditional radiography in multiple studies.^34^, ^35^ These imaging techniques allow for the identification of subtle bony lesions and soft tissue abnormalities that may not be visible on radiographs. While incorporating CT as a reference standard could have further validated the effectiveness of the LM/OB combination and provided a clearer understanding of its limitations, the low number of cases in our study population that underwent concurrent CT examination prevented this approach. Additionally, histopathological examination could have offered insights into the underlying tissue changes corresponding to the radiographic findings, enhancing the interpretation of mild or equivocal lesions. Future studies incorporating these advanced diagnostic methods would not only improve the precision of pathology detection but also clarify the clinical relevance of radiographic findings in the context of long‐term soundness and performance outcomes.

Another limitation of this study is the composition of the horse population examined. Most horses were presented due to lameness, with a small subset comprising sound horses undergoing pre‐purchase examinations (PPE). Typically, horses subjected to radiographic evaluation during PPEs have undergone prior clinical examinations confirming their soundness. Radiographic findings can vary significantly between lame and sound horses. A retrospective study analysing PPEs found that 52.8% of the evaluated horses exhibited lameness, and the prevalence of radiographic abnormalities was higher in these horses compared to their sound counterparts.^36^ This suggests that the inclusion of a predominantly lame population in our study could skew the prevalence and types of pathologies detected, potentially limiting the applicability of our results to sound horses typically assessed during PPEs. While this may not fully reflect the population of horses undergoing PPE, the inclusion of lame horses increased the number of detectable findings, allowing for a more comprehensive assessment of radiographic abnormalities and their visibility across different view combinations.

Despite the overall higher prevalence of radiographic abnormalities in this study, the low occurrence of certain specific pathologies, such as osseous cyst‐like lesions, presents an additional limitation. A lower prevalence of these lesions reduces the statistical power to detect true differences between radiographic projection combinations. Osseous cyst‐like lesions, though relatively uncommon, are clinically significant due to their potential association with joint lameness and future soundness concerns, depending on their size, location, and activity.^37^ With small sample sizes for specific conditions, the risk of Type II errors increases, making it difficult to identify meaningful differences or trends. However, the study still provides valuable insights into more commonly encountered pathologies, and future research could build on these findings by including larger or targeted populations with a higher prevalence of specific conditions.

Only two observers with different degrees of radiological training were used in this study, reflecting a focus on inter‐ and intra‐observer agreement within the discipline. While this design is common in agreement studies, the inclusion of a third observer from a different clinical background, such as a highly experienced equine orthopaedic clinician, could have added valuable perspective and improved generalisability. Future studies may benefit from evaluating cross‐disciplinary agreement to better reflect real‐world interpretation variability.

## 5 CONCLUSION

This study demonstrated that the LM/OB combination provides a practical and efficient alternative to the full radiographic series for many routine PPE scenarios. It strikes a balance between diagnostic reliability, time efficiency, and reduced radiation exposure, making it suitable for detecting conditions such as fragmentation and sesamoid bone fractures. However, clinicians should remain cautious when detecting certain pathologies, such as subchondral bone changes, as these may require further projections or advanced imaging. A more tailored approach, considering the horse's age and discipline, may therefore be warranted. Broader validation and integration of advanced imaging may further refine PPE protocols and improve outcome prediction.

## FUNDING INFORMATION

There was no external or internal funding for this study.

## CONFLICT OF INTEREST STATEMENT

The authors declare no conflicts of interest.

## AUTHOR CONTRIBUTIONS

**A. Northwood:** Writing – original draft; investigation; conceptualization; methodology; writing – review and editing; project administration; validation; formal analysis; resources; data curation. **D. Berner:** Conceptualization; investigation; writing – original draft; methodology; writing – review and editing; supervision; project administration; validation; formal analysis; resources; data curation.

## DATA INTEGRITY STATEMENT

A. Northwood had full access to all the data in the study and takes responsibility for the integrity of the data and the accuracy of data analysis.

## ETHICAL ANIMAL RESEARCH

Research ethics committee oversight not required by this journal: Retrospective study of clinical records.

## INFORMED CONSENT

Explicit owner consent for inclusion of animals in this study was not obtained. Owners/trainers were made aware that case information may be used for research in general.

## Data Availability

The data that support the findings of this study are openly available in Figshare at https://doi.org/10.6084/m9.figshare.29855252.v1.
